# Life in a Contaminated Environment: How Soil Nematodes Can Indicate Long-Term Heavy-Metal Pollution

**DOI:** 10.2478/jofnem-2022-0053

**Published:** 2022-11-20

**Authors:** Marek Renčo, Andrea Čerevková, Jakub Hlava

**Affiliations:** 1 Institute of Parasitology, Slovak Academy of Sciences, Košice, Slovak Republic; 2 Department of Zoology and Fisheries, Czech University of Life Sciences Prague, Prague Suchdol, Czech Republic

**Keywords:** bioindicators, ecology, environmental impact, interaction, heavy metals, pollution, soil nematodes

## Abstract

We investigated the genera, trophic groups, and functional guilds of soil nematodes at five alluvial meadows along the Litavka River in the Czech Republic to assess their usefulness as indicators of heavy metal pollution in soils. The Litavka River flows around the waste-sedimentation pond of a smelter in the city of Příbram in the Central Bohemian Region. Lead, zinc, and arsenic are the main pollutants in the soils in the vicinity of the smelter. The alluvial meadows closest to the pond and mine waste were the most heavily polluted sites, and contamination decreased downstream along the river with increasing distance from the sources of pollution. The nematode communities were sensitive to pollution, with the most contaminated sites having considerably fewer nematode individuals, fewer genera, and a less diverse and more degraded food web with less nematode biomass. Arsenic, lead, and zinc contents were significantly negatively correlated with the numbers of bacterivores, predators, omnivores, plant parasites, and fungivores, which were significantly less abundant at highly polluted sites. This correlation suggests that nematode groups with higher c-p values, and those with c-p 1 and 2 designations, can be useful indicators of high heavy-metal contamination in areas polluted for a long time. In contrast, the abundance of c-p 3 plant parasitic nematodes was positively correlated with copper, nickel, and zinc contents and with soil-moisture content in the alluvial meadows. Maturity index (MI) and MI2-5 were the most sensitive indices of the degree of disturbance of the soil ecosystem, with enrichment index, structure index, and basal index indicating the altered decomposition channels and diminished structure of the food web.

Heavy metals are extremely persistent in the environment; they are not biodegradable and not degradable by heat and thus readily accumulate to toxic levels, mainly due to long-term anthropogenic activities (Sharma *et al*., 2007). The Příbram region is one of the most affected areas by heavy-metal contamination in the Czech Republic, caused mainly by the mining and smelting industries (Mayerová *et al*., 2017). Borůvka *et al*. (1996) reported that three sources of soil pollution could be distinguished in this area: naturally increased metal content due to the specific composition of the parental rocks, atmospheric deposition from a smelter, and flooding with water from the Litavka River polluted with metal-processing wastes.

As reported by Kašpárek (1984), the Litavka River frequently floods. Large floods (water flows >55 m^3^ · s^−1^) occurred here 27 times between 1931 and 2007 (unpublished data from the Czech Hydrometeorological Institute). Ponds that hold and sediment mine waste are close to the Litavka River, so their walls have been breached several times by these floods, and the contents of the ponds, containing very high amounts of heavy metals and other risk elements, have been released into the river water and to the alluvia. Therefore, the soils in the alluvia are extremely polluted (Borůvka *et al*., 1996). This pollution covers a narrower strip of land compared to the pollution caused by atmospheric deposition, but the level of pollution is extremely high.

Several studies have assessed the accumulation and/or contents of heavy metals in the floodplain soils around the Litavka River (Borůvka *et al*., 1996; Žák *et al*., 2009; Dlouhá *et al*., 2013; Kebonye *et al*., 2021). Such traditional analytical approaches measure the contents of total or extractable heavy metals and provide information on their general distribution but not on the biological impacts they may have on an ecosystem. These data may be of limited use for assessing environmental importance (Paton *et al*., 2006). Bioassays that use soil organisms with specific reactions to changes in the environment are therefore needed to complement conventional chemical quantifications of heavy metals and to provide data on their toxicity (Campbell *et al*., 1997; Chapman *et al*., 2013; Fajardo *et al*., 2019). The abundance and diversity of soil nematodes respond quickly and quantifiably to various changes in the environment (Bongers and Ferris, 1999; Zhao and Neher, 2013; Renčo *et al*., 2015). Nematodes have several appropriate properties of bioindication: (1) they are ubiquitous and are thus adapted to a wide range of environmental conditions, (2) they are represented by all known trophic groups, and (3) they are sufficiently translucent for their diagnostic internal features to be seen without dissection and their life cycles are relatively short (Bongers and Ferris, 1999). Soil nematodes are also small and restricted to their living space, so they have limited ability to leave their habitat when conditions change. Soil nematodes are aquatic organisms as their survival depends strongly on the availability of free water for their activity, so they are in direct contact with dissolved substances.

Relationships between heavy-metal contamination and communities of soil nematodes have attracted increasing attention in recent years (Li *et al*., 2006; Shao *et al*., 2008; Park *et al*., 2011; Šalamún *et al*., 2012; Gutiérrez *et al*., 2016; Martinez *et al*., 2018). We conducted the first survey of the communities of soil nematodes in the ecosystem of alluvial meadows along the Litavka River, where we expected heavy-metal contamination from the waste-sedimentation ponds of a smelter near the city of Příbram (Dlouhá *et al*., 2013). We hypothesized that nematode abundance, species richness, taxonomic diversity, and trophic complexity would be low as a direct response to elevated levels of heavy metals. We also hypothesized that metal content would decrease with increasing distance from the source of pollution downstream along the river, with correspondingly higher nematode abundances, species richness, diversity, and trophic structuring.

## Material and Methods

### Site description and soil sampling

**Figure 1:**
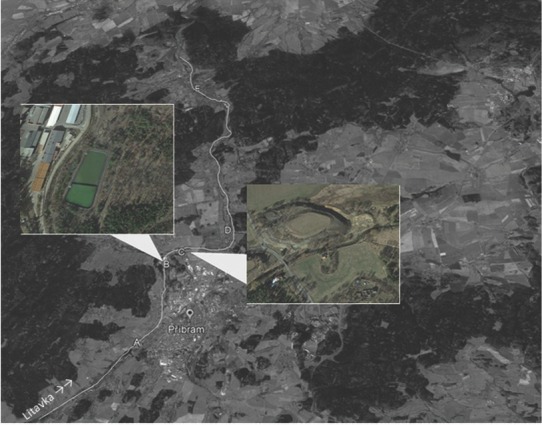
Map of selected localities: (A) control meadows situated 3.6 km before pollution source; (B) meadow near waste sedimentation pond; (C) meadow near the heap of mine waste situated 680 m from (B); and (D) meadows near old bakery mill situated 3.7 km from (B) and (E) meadow close to village Jince situated 13.3 km from the pollution source.

This study was performed in five alluvial meadows along the stream of the Litavka River as linear sources of pollution from a waste-sedimentation pond near the river. Selected sites were: (A) a control meadow 3.6 km upstream of the source of pollution (49°40¢N, 13°58¢E), (B) a meadow near the waste-sedimentation pond (49°42¢N, 13°59¢E), (C) a meadow near a mining Terril 680 m downstream from site B (49°42¢N, 13°59¢E), (D) a meadow near an old bakery mill 3.7 km downstream from (B) (49°43¢N, 14°07¢E), and (E) a meadow close to the village of Jince 13.3 km downstream from the source of pollution (49°47¢N, 13°59¢E) (Fig. 1). The river has a gradient of approximately 5 m/km and a single active channel, with no artificial source of pollution upstream before the control site. The river valley has a typical width of 100 m to 200 m and is relatively flat with abundant ditches (Faměra *et al*., 2018). Soil samples were collected in October 2021 from the surface horizon (0–20 cm) of each meadow using quadrat sampling (Renčo and Čerevková, 2015). Four replicates, consisting of five mixed subsamples, were collected from each site.

### Soil physicochemical analyses

Before the analysis, soil samples were air-dried, pounded, and sifted through a 0.2 mm sieve. Soil acidity (pH KCl) was determined using a HI2031B pH electrode meter (Hanna Instruments, Voehringen, Baden-Wuerttemberg, Germany) in the suspension of soil (10 g) and soil sample preparation solution HI7051L (25 ml) at room temperature. Soil moisture of the replicates was measured gravimetrically as the loss of weight after drying the 100 g of soil to a constant weight at 105°C for 12 hr. The contents of organic carbon (C) and total nitrogen (N) were determined from 0.25 g of soil using a Vario MACRO Elemental Analyzer (CNS Version; Elementar Analysensysteme, Langenselbold, Germany). Organic C content was determined based on the difference between total C and C bound in carbonates. The pseudo-total contents of elements in the 0.25 g of soil were determined by decomposing the samples using pressurized wet ashing (Ethos 1 microwave-assisted wet-digestion system, Milestone, Leutkirch im Allgau, Germany) as described by Fröhlichová *et al*. (2018). The proportion of bioaccessible risk element content in the soils was determined in the 0.5 g of soil sample as described by Quevauviller *et al*. (1993) using 0.11 mol · L^−1^ CH_3_COOH as an extracting agent in ratio 1:40 (w/v). Each extraction was carried out in triplicate. The supernatant was separated using a Hettich Universal 30 RF centrifuge (Hettich, Taufkirchen, Germany) at 3,000 rpm (460×*g*) for 10 min.

## Nematode extraction, identification, and community analyses

A combination of Cobb sieving and decanting (Cobb, 1918) and a modified Baermann technique (van Bezooijen, 2006) were used for extracting nematodes from 100 g of fresh soil in aqueous soil suspensions using a set of two cotton-propylene filters. One or two filter trays were used for each sample to prevent material from exceeding 0.5 cm in depth above the filter. Aqueous suspensions were removed after 48 hr of extraction at room temperature, and nematodes were counted under a stereomicroscope (40× magnification). Excessive water was removed, and the nematodes were fixed with a hot solution of 4% formaldehyde and 1% glycerol (Seinhorst, 1962). At least 100 nematodes were identified at the species level based on the original species descriptions and accessible taxonomic keys of nematode genera and groups using an Eclipse 90i light microscope (Nikon, Tokyo, Japan) at standard magnifications.

Nematode abundance in each sample was expressed as the number of individuals per 100 g of dry soil. Nematode genera were assigned to trophic groups as described by Yeates *et al*. (1993), adjusted and supplemented by following Sieriebriennikov *et al*. (2014), and assigned to 17 functional guilds integrating nematode feeding strategies (trophic groups) and the nematode colonizer-persister (c-p) scale (Bongers and Bongers, 1998). The six nematode trophic groups were: bacterivores (Ba), fungivores (Fu), predators (P), omnivores (Om), plant parasites (Pp), and insect parasites. The Pp group included both obligatory and facultative plant parasites that may attack plants or fungi. Colonizer-persister groups characterizing nematode life strategies are classified on a scale of 1 to 5 (Bongers, 1990). C-p1 represents “r-strategists” (colonizers), with short life cycles, small eggs, high fecundity, high colonization ability, and high tolerance to disturbance, eutrophication, and anoxybiosis. Colonizers generally live in ephemeral habitats. At the other end of the scale, c-p5 nematodes represent “k-strategists” (persisters), with the longest generation times, largest bodies, lowest fecundities, and the highest sensitivity to disturbance. Persisters are never dominant in a sample and generally live in stable habitats (Bongers, 1990). C-p scaling allows the calculation of the basal maturity index (MI) for non-parasitic nematodes, the plant-parasitic index (PPI) for plant parasites only (Bongers, 1990), and the MI2–5 MI (Yeates, 1994) for c-p2-5 nematode taxa. Functional guilds allow the calculation of the enrichment index (EI), the structure index (SI), the basal index (BI), and the channel index (CI) proposed by Ferris *et al*. (2001). The genus diversity index (H´gen) defined by Shannon and Weaver (1949) was also calculated. All indices (except H´gen) and total biomass were calculated using the NINJA online program (Sieriebriennikov *et al*., 2014); (https://shiny.wur.nl/ninja/), including graphic depictions of the conditions of the soil food webs, the “faunal profile” (Ferris *et al*., 2001), which was modeled separately for each sample, and the functional metabolic footprint (Ferris, 2010), which was modeled for each site.

### Statistical analysis

Statistical analyses were done using STATISTICA version 14.0 (TIBCO, 2020) and CANOCO 5 for Windows version 5 (Ter Braak and Šmilauer, 2012). All response variables were subjected to a one-way ANOVA to determine the overall effect of heavy-metal content on the nematode communities, after checking the homogeneity of variance using Levene’s test. When necessary, data were log(x + 1) transformed. Tukey’s honestly significant difference (HSD) was applied to identify significant differences in the variables between sites at *P* < 0.05. Nonparametric Spearman’s correlation coefficient was calculated to identify the relationships between the characteristics of the nematode communities and the heavy-metal contents at the study sites. Correlations at *P* < 0.05 and *P* < 0.01 were considered significant.

A redundancy analysis (RDA) was used on the main nematode genera associated with the alluvial meadows, with the heavy-metal contents as explanatory variables, to identify the relationships between the nematodes and trace elements. All data were log-transformed before use. The effects of the explanatory variables were quantified by automatic forward selection.

## Results

### Soil physicochemical properties and heavy-metal contents

The soils at sites A and E had neutral pH of 6.89 and 6.75, respectively. The soils at sites B, C, and D were moderately acidic, with pH ranging from 5.20 to 5.50, which differed significantly from the control site (A) to site E, the meadow farthest from the source of pollution (*P* < 0.05). The N and C contents also differed significantly among the sampling sites (*P* < 0.05). The soil N content ranged from 0.61% to 0.87% among the meadows and was lowest at site B. The soil C content was significantly lower at sites B and C compared with the control site and sites D and E (*P* < 0.05). Average soil-moisture content ranged between 29.5% and 35.2% but did not differ significantly among the sampling sites (Table 1).

**Table 1:** Soil physico- chemical properties (mean ± SD) associated with alluvial meadows in the vicinity of the Příbram mine along the Litavka River.

Parameter	Sites				
	A	B	C	D	E
pH/KCl	6.89 ± 0.25^a^	5.42 ± 0.21^a^	5.20 ± −0.11^b^	5.56 ± 0.16^b^	6.75 ± 0.18^a^
Soil moisture (%)	35.2 ± 5.6^a^	31.5 ± 2.9^a^	29.5 ± 3.1^a^	30.5 ± 3.3^a^	31.5 ± 2.8^a^
N_tot_ (%)	0.87 ± 0.15^a^	0.61 ± 0.10^b^	0.74 ± 0.32^b^	0.66 ± 0.15^b^	0.83 ± 0.22^a^
C_ox_ (%)	14.25 ± 1.69^a^	8.25 ± 2.19^b^	10.67 ± 3.61^b^	12.29 ± 2.69^a^	12.87 ± 3.16^a^

Means followed by the same letters on the same rows are not statistically different by Tukey's honestly significant difference (HSD) at *p* < 0.05, *n* = 4.

All heavy-metal contents in the soil samples were significantly higher (*P* < 0.05) at sites B and C than at the control meadow (site A) (except for nickel (Ni) and chromium (Cr) at site C). Arsenic (As), lead (Pb), and zinc (Zn) contents were 18.1, 6.8, and 8.5 × higher at site B; 17.8, 7.3, and 19.8 × higher at site C; and 4.6, 4.9, and 11.1 × higher at site D than at site A, respectively. Pb and Zn contents were highest at site C, and the As content was highest at site B but decreased downstream with increasing distance from the source of pollution. The heavy-metal contents, however, were still higher than the regulatory limits, even in the most distant meadow (E) and at the control site (A), especially for As, Pb, and Zn (Table 2).

**Table 2:** Total concentrations of heavy metals (mg/kg) (mean ± SD) associated with alluvial meadows in the vicinity of the Příbram mine along the Litavka River.

Element	Sites					Max
	A	B	C	D	E	
As	38.2 ± 3.3^a^	688.8 ± 91.8^c^	677.4 ± 181.1^c^	176.2 ± 47.7^b^	31.8 ± 17.8^a^	20
Cd	1.9 ± 0.2^a^	19.5 ± 1.9^b^	51.5 ± 23.1^c^	28.4 ± 12.9^bc^	2.1 ± 1.0^a^	0.5
Cr	37.9 ± 5.1^a^	58.2 ± 1.3^b^	68.8 ± 59.9^a^	40.4 ± 5.7^a^	28.7 ± 1.7^a^	90
Cu	35.7 ± 5.3^a^	97.6 ± 8.7^b^	139.6 ± 24.6^c^	61.9 ± 30.7^ab^	23.1 ± 11.9^a^	60
Ni	19.1 ± 1.3^a^	38.1 ± 2.4^b^	33.2 ± 8.2^ab^	22.9 ± 8.3^a^	15.4 ± 1.8^a^	50
Pb	553.3 ± 237.6^a^	3734.1 ± 1915.6^b^	4030.9 ± 165.1^bc^	2682.2 ± 460.3^b^	689.8 ± 474.3^a^	60
Zn	346.2 ± 34.6^a^	2949.5 ± 435.9^b^	6867.8 ± 1374.9^c^	3866.4 ± 1146.2^b^	566.4 ± 490.1^a^	120

Means followed by the same letters on the same rows are not statistically different by Tukey's honestly significant difference (HSD) at *p* < 0.05, *n* = 4. Max – limits posted by The Decree of the Ministry of Land Management of the Czech Republic No. 437/2016 on the admissible values of harmful substances in uncontaminated soil.

### Nematode abundance, genera, and diversity

The average nematode abundance ranged from 513 to 2,193 individuals per 100 g of dry soil. Abundance was highest at site A and lowest at site C (Table 3). The most polluted sites (B, C, and D) had significantly fewer nematodes compared with site A (*P* < 0.05), and abundance increased from site C to E. Total nematode biomass, the diversity index, H´, MI, and SI in the soil samples had similar patterns.

**Table 3:** Total nematode abundance, number of genera, nematode community indices associated with alluvial meadows in the vicinity of the Příbram mine along the Litavka River (mean ± SD).

Indices	A	B	C	D	E
Nematode abundance	2193.2 ± 358.9^a^	595.8 ± 130.9^b^	513.8 ± 122.3^b^	974.9 ± 340.4^b^	1674.1 ± 263.5^a^
Genera number	31.8 ± 1.7^a^	25.8 ± 1.5^b^	27.5 ± 5.1^a^	21.5 ± 3.1^b^	30.5 ± 4.2^a^
Maturity Index	2.68 ± 0.12^a^	2.12 ± 0.09^b^	2.20 ± 0.10^b^	2.22 ± 0.21^b^	2.63 ± 0.15^a^
Maturity Index (2-5)	2.89 ± 0.07^a^	2.33 ± 0.16^b^	2.37 ± 0.15^b^	2.33 ± 0.19^b^	2.85 ± 0.22^a^
Plant Parasitic Index	2.69 ± 0.11^a^	2.80 ± 0.18^a^	2.88 ± 0.04^a^	2.77 ± 0.14^a^	2.73 ± 0.16^a^
Diversity Index (H´gen)	3.49 ± 0.15^a^	2.13 ± 0.25^b^	2.47 ± 0.26^b^	2.22 ± 0.18^b^	3.09 ± 0.10^a^
Channel Index	8.8 ± 4.0^a^	23.0 ± 11.8^b^	36.4 ± 12.9^b^	30.9 ± 6.6^b^	22.9 ± 19.6^ab^
Basal Index	16.6 ± 2.3^a^	32.9 ± 8.3^b^	33.9 ± 10.7^b^	33.7 ± 4.6^b^	19.9 ± 9.3^a^
Enrichment Index	71.5 ± 4.4^a^	53.2 ± 10.9^b^	50.3 ± 11.5^b^	37.9 ± 16.2^b^	66.7 ± 10.1^b^
Structure Index	72.0 ± 3.2^a^	44.5 ± 18.7^b^	49.8 ± 13.1^b^	47.9 ± 16.2^b^	73.8 ± 12.3^a^
Total biomass, mg	7.5 ± 1.2^a^	2.1 ± 1.2^b^	1.9 ± 1.3^b^	4.9 ± 2.9^b^	8.6 ± 2.5^a^

Means followed by the same letters on the same rows are not statistically different by Tukey's honestly significant difference at *P* < 0.05, *n* = 4.

**Figure 2:**
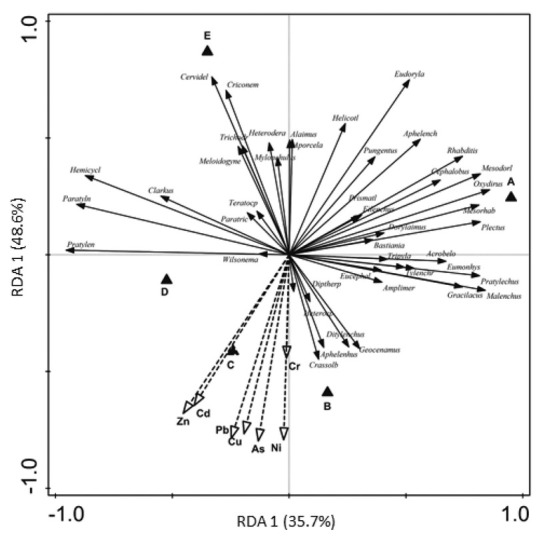
RDA biplot of prevailing nematode genera and heavy metals contents in the five alluvial meadows along the Litavka River in the vicinity of the Příbram mine pollution sources. (A) a control meadow 3.6 km upstream of the source of pollution; (B) meadow near waste sedimentation pond; (C) meadow near the heap of mine waste situated 680 m from (B); and (D) meadows near old bakery mill situated 3.7 km from (B) and (E) meadow close to village Jince situated 13.3 km from the pollution source. *Amplimer* = *Amplimerlinius; Acrobelo* = *Acrobeloides; Aphelench* = *Aphelenchoides; Aporcela* = *Aporcelaimellus; Crassolb* = *Crassolabium; Criconem* = *Criconemoides; Dipttherp* = *Diptherophora; Eucephal* = *Eucephalobus; Eudoryla* = *Eudorylaimus; Eumonhys* = *Eumonhystera; Helicotl* = *Helicotylenchus; Hemicycl* = *Hemicycliophora; Heterocp* = *Heterocephalobus; Mesodorl* = *Mesodorylaimus; Mesorhab* = *Mesorhabditis; Paratyln* = *Paratylenchus; Pratylech* = *Pratylenchoides; Prismatl* = *Primatolaimus; Teratocep* = *Teratocepahlobus; Trichodr* = *Trichodorus; Tylenchr* = *Tylenchorhynchus*. RDA, redundancy analysis.

A total of 78 nematode genera were recorded in the alluvial meadows downstream along the Litavka River. Of these genera, 25 were bacterivores (Ba), 8 were fungivores (Fu), 11 were omnivores (Om), 7 were predators (P), 26 were plant parasites (Pp), and 1 was an insect parasite (In) (Table 4). The genera *Acrobeloides*, *Cephalobus*, *Mesorhabditis*, *Plectus*, and *Rhabditis* were the most abundant bacterivores; *Aphelenchoides* and *Dorylaimoides* were the most abundant fungivores; *Eudorylaimus* and *Mesodorylaimus* were the most abundant omnivores; *Oxydirus* was the most abundant predator; and *Helicotylenchus*, *Heterodera*, and *Malenchus* were the most abundant plant parasites at site A (control). The majority of nematode genera were generally less abundant under the high heavy-metal loads at sites B, C, and D. The RDA of the heavy-metal contents and the prevailing nematode genera explained 35.7% of the variations (RDA1) and identified a significant negative correlation between the abundance of the majority of the genera and heavy-metal pollution at sites B, C, and D (Fig. 2). The abundance of several nematode taxa, e.g., *Acrobeloides*, *Cephalobus*, and *Cervidellus* (Ba); *Aphelenchoides* (Fu); *Aporcelaimellus* and *Eudorylaimus* (Om); and *Mylonchulus* (P), increased slightly with increasing distance from the sources of pollution. In contrast, the plant-parasitic genera *Geocenamus*, *Helicotylenchus*,

**Table 4:** A abundance of nematode genera (mean ± SD) associated with alluvial meadows in the vicinity of the Příbram mine along the Litavka River.

Genus/trophic group	c-p	A	B	C	D	E
**Bacterivores**						
*Mesorhabditis*	1	251.6 ± 103.4	24.3 ± 13.0	22.6 ± 14.2	5.3 ± 8.3	24.8 ± 14.5
*Plectus*	2	241.3 ± 44.1	23.9 ± 20.7	10.5 ± 2.9	0.9 ± 0.5	15.4 ± 2.8
*Rhabditis*	1	187.0 ± 63.9	21.8 ± 17.7	5.9 ± 1.2	9.5 ± 11.6	26.3 ± 10.8
*Acrobeloides*	2	143.3 ± 75.1	72.1 ± 8.4	15.4 ± 7.9	51.1 ± 26.6	40.5 ± 35.6
*Cephalobus*	2	123.5 ± 61.0	12.1 ± 8.9	5.9 ± 3.9	9.6 ± 10.2	23.2 ± 18.7
*Eucephalobus*	2	53.3 ± 27.8	12.2 ± 9.3	20.5 ± 10.8	22.5 ± 15.9	11.6 ± 10.1
*Prismatolaimus*	3	51.1 ± 60.8	12.1 ± 7.9	7.0 ± 3.3	11.9 ± 17.5	17.6 ± 5.3
*Bunonema*	1	27.4 ± 14.9	-	-	-	2.2 ± 4.4
*Eumonhystera*	2	26.2 ± 24.1	4.6 ± 5.9	5.0 ± 2.4	-	4.4 ± 8.7
*Heterocephalobus*	2	18.3 ± 5.6	4.5 ± 3.4	-	8.6 ± 3.9	-
*Alaimus*	4	9.1 ± 18.1	0.6 ± 1.1	4.3 ± 7.3	-	12.2 ± 9.8
*Bastiania*	3	8.7 ± 5.9	2.1 ± 4.2	2.6 ± 1.9	1.9 ± 3.8	4.6 ± 9.3
*Anaplectus*	2	8.0 ± 5.4	1.9 ± 1.4	-	-	-
*Cervidellus*	2	7.9 ± 15.8	-	-	8.1 ± 6.6	47.0 ± 12.8
*Teratocephalus*	3	2.3 ± 4.6	1.4 ± 2.9	9.5 ± 2.6	1.9 ± 3.8	10.3 ± 5.9
*Eudiplogaster*	2	1.5 ± 3.0	0.5 ± 1.0	-	1.9 ± 3.8	2.3 ± 4.6
*Acrobeles*	2	-	-	-	1.9 ± 3.8	16.8 ± 11.5
*Amphidelus*	4	-	-	1.9 ± 3.8	-	-
*Ceratoplectus*	2	-	1.1 ± 2.3	2.2 ± 2.5	-	-
*Diploscapter*	1	-	1.4 ± 1.7	-	-	2.2 ± 4.4
*Euteratocephalus*	2	-	4.0 ± 3.8	3.0 ± 2.0	-	-
*Chiloplacus*	2	-	-	-	2.9 ± 3.7	8.6 ± 10.6
*Panagrolaimus*	1	-	1.0 ± 2.1	-	-	2.2 ± 4.4
*Prodesmodora*	3	-	1.5 ± 2.0	-	-	-
*Wilsonema*	2	-	15.4 ± 14.8	-	1.9 ± 2.1	12.4 ± 8.3
**Fungivores**						
*Dorylaimoides*	4	104.9 ± 59.4	-	-	-	-
*Aphelenchoides*	2	113.6 ± 54.2	7.9 ± 5.9	8.2 ± 6.6	9.5 ± 11.5	39.6 ± 7.8
*Aphelenchus*	2	24.4 ± 37.3	20.9 ± 12.3	2.1 ± 3.0	11.9 ± 6.2	2.2 ± 4.4
*Ditylenchus*	2	12.5 ± 15.4	9.3 ± 7.6	6.5 ± 3.0	2.9 ± 5.9	3.2 ± 6.5
*Diptheropthora*	3	10.9 ± 14.9	5.0 ± 5.0	8.5 ± 4.5	6.2 ± 2.8	6.0 ± 4.2
*Tylencholaimus*	4	5.4 ± 6.3	4.2 ± 8.3	-	-	7.6 ± 10.2
*Tylencholaimellus*	4	4.5 ± 9.1	0.5 ± 1.0	-	-	-
*Paraphelenchus*	2	-	1.2 ± 2.5	-	-	1.6 ± 3.2
**Omnivores**						
*Eudorylaimus*	4	91.1 ± 61.5	3.0 ± 3.9	0.9 ± 0.9	2.9 ± 3.7	53.1 ± 38.5
*Mesodorylaimus*	4	76.2 ± 26.6	-	-	-	4.4 ± 8.7
*Oxydirus*	5	60.5 ± 40.2	0.8 ± 1.6	-	-	1.6 ± 3.2
*Dorylaimus*	4	24.3 ± 40.7	0.5 ± 1.0	2.4 ± 1.8	-	1.6 ± 3.2
*Aporcelaimellus*	5	13.9 ± 16.4	1.0 ± 2.1	2.0 ± 1.4	3.8 ± 4.1	21.1 ± 6.9
*Pungentus*	4	10.7 ± 12.6	0.4 ± 0.8	0.5 ± 1.0	-	4.9 ± 5.7
*Achromadora*	3	3.1 ± 6.1	-	-	-	-
*Crassolabium*	4	1.5 ± 3.0	6.7 ± 6.3	1.5 ± 1.9	1.9 ± 1.2	-
*Axonchium*	5	-	2.8 ± 5.7	-	-	-
*Discolaimium*	4	-	2.1 ± 4.2	-	-	-
*Microdorylaimus*	4	-	-	0.5 ± 1.1	9.5 ± 11.4	-
*Paractinolaimus*	5	-	0.4 ± 0.8	-	-	-
**Predators**						
*Tripyla*	3	7.7 ± 9.1	2.9 ± 5.9	-	1.9 ± 3.8	-
*Mononchus*	4	7.3 ± 9.0	0.5 ± 1.0	3.4 ± 2.1	-	-
*Prionchulus*	4	6.2 ± 8.7	-	-	-	1.6 ± 2.1
*Clarkus*	4	3.1 ± 6.1	0.9 ± 1.0	8.0 ± 4.6	8.1 ± 5.0	14.4 ± 9.8
*Mylonchulus*	4	3.1 ± 6.2	3.7 ± 5.9	1.1 ± 2.1	-	17.3 ± 14.6
*Nygolaimus*	5	-	-	-	-	2.2 ± 4.4
**Plant parasites**						
*Helicotylenchus*	3	163.8 ± 82.9	38.7 ± 31.1	60.9 ± 21.6	85.7 ± 64.8	118.0 ± 57.6
*Heterodera*	3	57.9 ± 47.4	31.8 ± 2.4	27.8 ± 17.3	78.6 ± 21.8	52.3 ± 134.8
*Pratylenchoides*	3	36.9 ± 30.1	5.0 ± 1.2	-	-	-
*Malenchus*	2	50.9 ± 52.7	9.2 ± 2.5	5.8 ± 2.4	95.5 ± 21.9	2.2 ± 4.4
*Rotylenchus*	3	29.3 ± 31.7	-	0.5 ± 1.1	0.9 ± 1.9	-
*Geocenamus*	3	27.0 ± 20.3	67.7 ± 47.0	62.5 ± 16.3	53.8 ± 4.4	22.4 ± 16.6
*Gracilacus*	2	26.6 ± 12.9	39.8 ± 33.0	-	-	7.0 ± 13.9
*Filenchus*	2	15.3 ± 12.0	5.1 ± 4.7	0.9 ± 1.9	4.3 ± 3.3	9.2 ± 10.6
*Tylenchorhynchus*	3	14.7 ± 12.0	23.9 ± 27.1	3.9 ± 5.1	-	4.9 ± 5.7
*Meloidogyne*	3	7.5 ± 8.2	10.7 ± 26.2	-	20.5 ± 14.5	99.6 ± 33.9
*Tylenchus*	2	6.8 ± 9.9	0.8 ± 1.6	0.5 ± 1.1	1.9 ± 3.8	-
*Coslenchus*	2	6.1 ± 12.2	2.8 ± 3.7	-	-	2.3 ± 4.6
*Amplimerlinius*	3	3.0 ± 6.1	1.3 ± 2.0	-	-	-
*Lelenchus*	2	2.6 ± 5.3	-	-	3.8 ± 7.6	-
*Criconemoides*	3	-	-	-	-	9.7 ± 7.0
*Hemicycliophora*	3	-	-	105.7 ± 49.2	303.1 ± 63.3	195.3 ± 110.3
*Longidorus*	5	-	3.4 ± 4.3	-	-	23.8 ± 8.2
*Mesocriconema*	3	-	-	1.6 ± 2.1	-	15.6 ± 12.1
*Paratrichodorus*	4	-	4.1 ± 1.5	-	-	24.9 ± 30.2
*Paratylenchus*	2	-	27.0 ± 16.2	33.3 ± 10.8	23.4 ± 9.7	177.1 ± 159.5
*Pratylenchus*	3	-	31.6 ± 8.4	45.1 ± 14.1	107.1 ± 28.9	122.8 ± 40.2
*Trichodorus*	4	-	4.3 ± 5.9	-	-	20.5 ± 18.4
*Xenocriconemella*	3	-	4.3 ± 7.2	-	-	-
*Aglenchus*	2	-	-	-	3.9 ± 2.5	-
*Boleodorus*	2	-	-	2.8 ± 3.5	-	-
*Psilenchus*	2	-	-	1.1 ± 1.2	-	-
**Insect parasites**						
*Steinernema*	1	-	-	2.8 ± 5.7	3.8 ± 4.4	

*Heterodera*, *Hemicycliophora*, and *Pratylenchus* were most abundant at sites B, C, and/or D (Table 4).

### Nematode trophic groups, functional guilds, community indices, and faunal profile

The nematode communities at site A (control) were characterized by the prevalence of bacterivores (Ba_1,2_), followed by plant parasites (Pp_2,3_), fungivores (Fu_2,4_), omnivores (Om_4_), and predators (P_5_) (Table 5). All bacterial functional guilds had significantly fewer individuals at sites B, C, and D than site A (*P* < 0.05), which were significantly negatively correlated with As, Pb, and Zn contents (*P* < 0.05, *P* < 0.01) (Table 6). The Spearman’s correlation analysis identified significant negative correlations between total nematode abundance, number of species, species diversity, Pb and Zn contents, and/or pH and copper (Cu) content (*P* < 0.05, *P* < 0.01) (Table 6). The abundances of predators (P_3,5_), omnivores (Om_4,5_), plant parasites (Pp_2_), and fungivores (Fu_2,4_) were strongly significantly correlated negatively with the Pb and Zn contents (*P* < 0.01) and were significantly less abundant at the highly polluted sites (Tables 5 and 6). The abundances of fungivores (Fu_2,3,4_) and omnivores (Om_5_) were negatively correlated with soil pH (*P* < 0.05). In contrast, the abundance of c-p 3 plant parasitic nematodes was significantly positively correlated with the Cu, Ni, Zn, and soil-moisture contents in the alluvial meadows (*P* < 0.05) (Table 6).

**Table 5:** Mean (±SD) abundance of nematode trophic groups and functional guilds associated with alluvial meadows in the vicinity of the Příbram mine along the Litavka River.

	A	B	C	D	E
**Bacterivores**	**1160.6** ± **604.5^a^**	**218.6** ± **56.3^b^**	**115.6** ± **44.9^b^**	**139.9** ± **56.8^b^**	**284.3** ± **119.9^b^**
Ba_1_	466.1 ± 142.5^a^	48.5 ± 31.2^b^	28.4 ± 18.3^b^	14.8 ± 10.2^b^	57.6 ± 30.0^b^
Ba^2^	623.3 ± 149.5^a^	152.3 ± 41.3^b^	61.9 ± 8.3^b^	109.3 ± 54.1^b^	182.3 ± 78.9^b^
Ba_3_	62.1 ± 60.6^a^	17.1 ± 13.8^b^	19.1 ± 15.3^b^	15.7 ± 25.1^b^	32.3 ± 27.2^ab^
Ba_4_	9.1 ± 18.1^a^	0.6 ± 1.1^b^	0.6 ± 1.1^b^	-	10.0 ± 11.7^a^
**Predators**	**87.6** ± **79.3**	**8.8** ± **6.8**	**12.4** ± **9.8**	**10.1** ± **6.1**	**37.1** ± **14.8**
P_3_	7.7 ± 9.2^a^	2.9 ± 5.8^a^	2.9 ± 5.8^a^	1.9 ± 3.8^a^	-
P_4_	19.5 ± 12.1^a^	5.1 ± 5.0^b^	12.4 ± 9.8^a^	8.1 ± 5.0^a^	33.3 ± 10.8^a^
P_5_	60.4 ± 40.2^a^	0.8 ± 1.6^b^	-	-	3.8 ± 4.5^b^
**Fungivores**	**276.1** ± **196.9^a^**	**49.0** ± **26.7^b^**	**25.3** ± **7.6^b^**	**30.6** ± **10.9^b^**	**60.6** ± **24.8^b^**
Fu_2_	150.3 ± 39.8^a^	39.4 ± 15.1^b^	16.8 ± 7.9^b^	24.3 ± 7.1^b^	46.6 ± 31.4^b^
Fu_3_	10.9 ± 14.9^a^	5.0 ± 5.0^a^	8.5 ± 7.9^a^	6.2 ± 6.5^a^	6.0 ± 8.2^a^
Fu_4_	114.8 ± 46.3^a^	4.6 ± 8.0^b^	4.6 ± 8.0^b^	-	7.6 ± 10.2^b^
**Omnivores**	**220.7** ± **167.4^a^**	**17.2** ± **6.6^b^**	**7.9** ± **3.0^b^**	**18.1** ± **19.0^b^**	**85.0** ± **44.3^ab^**
Om_3_	3.0 ± 6.1	-	-	-	-
Om_4_	203.6 ± 90.0^a^	12.7 ± 10.1^b^	5.9 ± 3.7^b^	14.3 ± 14.7^b^	63.9 ± 40.6^b^
Om_5_	14.0 ± 6.4^a^	4.3 ± 5.0^b^	2.0 ± 2.4^b^	3.8 ± 4.4^b^	21.1 ± 5.6^a^
**Plant parasites**	**451.8** ± **117.5^a^**	**299.7** ± **66.9^a^**	**351.4** ± **98.0^a^**	**782.5** ± **260.8^a^**	**893.8** ± **249.3^a^**
Pp_2_	108.3 ± 52.5^a^	84.6 ± 40.5^a^	44.4 ± 25.7^b^	187.3 ± 121.4^a^	397.7 ± 262.4^a^
Pp_3_	340.1 ± 15.7^a^	206.1 ± 67.4^a^	308.0 ± 84.9^a^	589.2 ± 206.3^a^	540.5 ± 111.2^a^
Pp_4_	-	8.4 ± 6.7	-	-	45.5 ± 35.8
Pp_5_	-	3.4 ± 4.3	-	-	23.8 ± 28.9

Means followed by the same letters on the same rows are not statistically different by Tukey’s HSD at *P* < 0.05, *n* = 4. Ba_1,2,3,4_ bacterivores; Fu_2,3,4_ fungivores; P_3,4,5_ predators; Om_3,4,5_ omnivores; Pp_2,3,4,5_ plant parasites. HSD, honestly significant difference.

Indices MI, MI2–5, SI, EI, and BI were significantly lower in the heavily polluted sites B, C, and D than in site A (*P* < 0.05) and consistently increased with increasing distance from the most polluted sites (Table 3). Total nematode biomass had a similar pattern, and PPI was balanced across all meadows. The contents of several trace elements were significantly correlated with some of the indices (Table 6). Pb and Zn contents were significantly (*P* < 0.01) negatively correlated with MI; Zn content was significantly negatively correlated with MI2–5 (*P* < 0.05); Pb content was significantly negatively correlated with EI (*P* < 0.05); and As, Pb, and Zn contents were significantly negatively correlated with SI (*P* < 0.05, *P* < 0.01).

**Figure 3:**
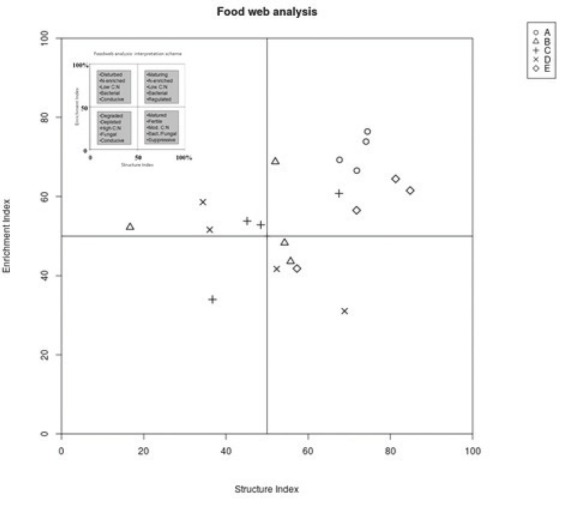
Plots of enrichment *vs*. structure indices associated with investigated alluvial meadows along the Litavka River contaminated by heavy metals in the vicinity of the Příbram mine pollution sources. A – control; (B) meadow near waste sedimentation pond; (C) meadow near the heap of mine waste situated 680 m from (B); and (D) meadows near old bakery mill situated 3.7 km from (B) and (E) meadow close to village Jince situated 13.3 km from the pollution source.

**Figure 4:**
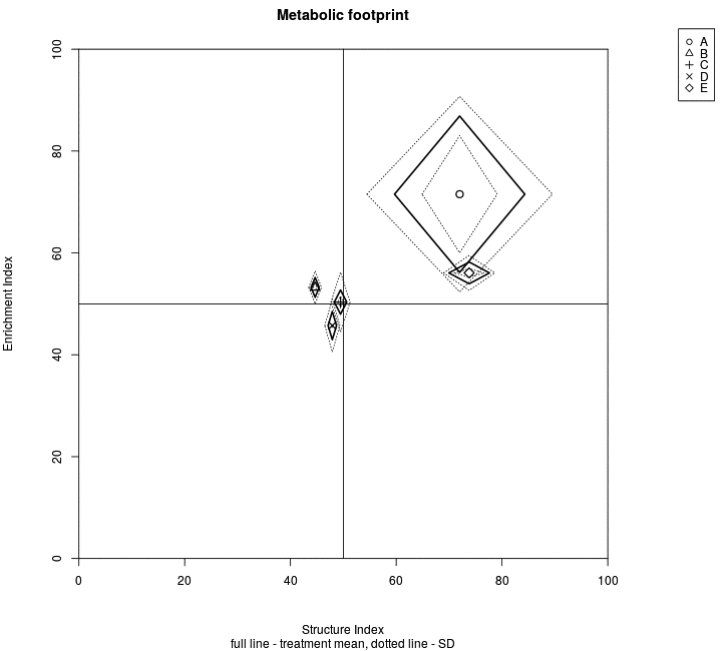
Functional metabolic footprints of nematode associated with investigated alluvial meadows along the Litavka River contaminated by heavy metals in the vicinity of the Příbram mine pollution sources. The vertical axis of each footprint represents the enrichment footprint and the horizontal axis represents the structural footprint. The *x*-axis coordinates of the metabolic footprint are calculated as SI – 0.5F*s*/*k* and SI + 0.5F*s*/*k*, where F*s* is the sum of standardized C utilization by structure indicator taxa. The *y*-axis coordinates are calculated as EI – 0.5F*e*/*k* and EI + 0.5 F*e*/*k*, where F*e* is the sum of standardized C utilization by enrichment indicator taxa. The functional metabolic footprint is depicted by sequentially joining points: SI – 0.5 F*s*/*k*, EI; SI, EI + 0.5 F*e*/*k*; SI + 0.5 F*s*/*k*, EI; SI, EI – 0.5 F*e*/*k*; and SI – 0.5 F*s*/*k*, EI. A – control; (B) meadow near waste sedimentation pond; (C) meadow near the heap of mine waste situated 680 m from (B); and (D) meadows near old bakery mill situated 3.7 km from (B) and (E) meadow close to village Jince situated 13.3 km from the pollution source.

The graphic representation of the “weighted faunal analysis” for the enrichment and structure indicators indicated that only the soil samples from sites A and E were mapped to quadrant B, which is characterized as a maturing soil food web, weakly to moderately disturbed, with the decomposition of organic matter controlled by both bacteria and fungi (Fig. 3). In contrast, the samples collected in the heavily polluted meadows were mapped to quadrats A and D, which represent soil food webs within a degraded and highly disturbed ecosystem. Similarly, the characteristics of the metabolic footprint of the nematode communities differed among the meadows (Fig. 4). The shape of the total functional footprint (total area) was similar to a square, and the footprint was largest at sites A and E, suggesting that the system was more metabolically balanced and stable than at the sites heavily polluted by heavy metals, which had rhomboid-shaped footprints.

## Discussion

The soil contents of hazardous elements in the area of our study were naturally higher than in other areas of the Czech Republic due to the specific composition of the parental rocks. The bedrocks consisted mainly of schists, sandstones, graywackes, and quartzes. These rocks were mostly covered in the alluvia by non-calcareous alluvial sediments. Minerals rich in potential risk elements include galenite (PbS), sphalerite (ZnS), boulangerite (Pb_5_Sb_4_S_11_), and antimonite (Sb_2_S_3_) (Borůvka and Vacha, 2006). These minerals are consistent with the data obtained from two of the alluvial meadows in our study. As, Pb, and Zn contents were relatively high (exceeding the admissible limits of harmful substances in soils) at control site A 3.6 km upstream from the main source of pollution and at site E 13.3 km downstream from the source.

Surveys conducted in the last two decades (Vaněk *et al*., 2005; Žák *et al*., 2009; Dlouhá *et al*., 2013; Kotková *et al*., 2019; Kebonye *et al*., 2021) have indicated that the floodplain area of the Litavka River was highly polluted by As, Pb, and Zn, which agrees with our records. The soils of the alluvial meadows in the vicinity of the waste-sedimentation ponds and mine-waste heaps had considerably higher amounts of Zn, As, and Pb compared with the control site and with the site farthest from the sources of pollution. The soils at these highly polluted sites in our study were also strongly acidic, consistent with the findings reported by Kebonye *et al*. (2021).

Our assumption that the differences in the nematode communities between our surveyed meadows could be attributed to the effects of Zn, Pb, and As contamination and higher soil acidity was thus reasonable. Nisa *et al*. (2021) reported that nematode abundance decreased from alkaline to acidic soils at 10 sites along the Kashmir Valley, which is also supported by our results. The meadows in our study heavily contaminated by Pb, Zn, and As from anthropic activities had acidic soils and significantly fewer nematodes and lower abundances of most nematode trophic groups and functional guilds relative to the meadows with neutral soil pHs and lower contents of hazardous elements. Similar findings were reported by Park *et al*. (2011) from very acidic (pH 2.8) soils polluted by Pb and Zn around an abandoned mine in South Korea and by Pen-Mouratov *et al*. (2008) from the Almalyk mining and metallurgical complex in the Republic of Uzbekistan. The soils in the study by Pen Mouratov *et al*. (2008), however, were weakly alkaline (pH 8.1) and polluted by Cu, Pb, and As, in contrast with the results of Nisa *et al*. (2021) and our observations. Pb and Zn contents were not correlated with the total number of nematodes in neutral soils (pH 6.8–7.1) in the tailings of the Baoshan Pb/Zn mine in China (Shao *et al*., 2008). Extremely alkaline soils (pH 9.0) heavily contaminated by magnesium (Mg) from the processing of Mg ore also had total nematode abundances similar to those from neutral soils less polluted by Mg (pH 6.9–7.4) and farther from the source of pollution (Šalamún *et al*., 2014).

**Table 6:** Spearman’s rank correlation coefficients between soil physico-chemical properties, heavy metals, and nematode parameters in the alluvial meadows in the vicinity of the Příbram mine along the Litavka River.

	As	Cd	Cr	Cu	Ni	Pb	Zn	pH/KCl	N_tot_	C_ox_	SM
Abundance	Ns	ns	ns	ns	ns	−0.862**	−0.748**	−0.659**	ns	ns	ns
No. genera	ns	ns	ns	ns	ns	ns	−0.451*	ns	ns	ns	ns
Ba_1_	−0.524*	ns	ns	ns	ns	−0.599*	−0.482*	ns	ns	ns	ns
Ba_2_	−0.633**	ns	ns	ns	ns	−0.826**	−0.859**	0.467*	0.561*	ns	ns
Ba_3_	−0.489*	ns	ns	ns	0.442*	−0.631**	−0.745**	ns	ns	ns	0.412*
Ba_4_	ns	ns	ns	ns	ns	−0.455*	ns	ns	ns	ns	ns
P_3_	−0.583*	ns	−0.409*	−0.460*	ns	−0.741**	−0.692**	ns	ns	ns	ns
P_4_	ns	ns	ns	ns	ns	ns	−0.423*	ns	ns	ns	ns
P_5_	ns	ns	ns	−0.559*	ns	−0.699**	−0.711**	ns	ns	ns	0.431*
Fu_2_	−0.507*	ns	ns	ns	ns	−0.853**	−0.626**	−0.492*	ns	ns	ns
Fu_3_	ns	ns	ns	ns	0.455*	ns	ns	−0.522*	ns	ns	ns
Fu_4_	−0.625**	ns	ns	−0.474*	ns	−0.769**	−0.834**	−0.439*	ns	ns	ns
Om_3_	ns	ns	ns	ns	ns	ns	ns	ns	ns	ns	ns
Om_4_	−0.569*	ns	ns	ns	ns	−0.845**	−0.869**	ns	−0.422*	ns	ns
Om_5_	−0.725**	ns	ns	ns	ns	−0.766**	−0.683**	−0.426*	−0.445*	ns	ns
Pp_2_	ns	ns	ns	ns	ns	−0.432*	−0.457*	ns	ns	ns	ns
Pp_3_	ns	ns	ns	0.466*	0.529*	ns	0.513*	ns	ns	ns	0.436*
Pp_4_	ns	ns	ns	ns	ns	ns	ns	ns	ns	ns	ns
Pp_5_	ns	ns	ns	ns	ns	ns	ns	ns	ns	ns	ns
H´spp	ns	ns	ns	−0.425*	ns	−0.722**	−0.634**	ns	ns	ns	ns
MI	ns	ns	ns	ns	ns	−0.584**	−0.605**	0.433*	ns	ns	ns
MI (2–5)	ns	ns	ns	ns	ns	ns	−0.552*	ns	ns	ns	ns
PPI	ns	ns	ns	ns	ns	ns	ns	ns	ns	ns	ns
CI	ns	ns	ns	ns	ns	ns	ns	ns	ns	ns	ns
EI	ns	ns	ns	ns	ns	−0.439*	ns	ns	ns	ns	ns
SI	−0.548*	ns	ns	ns	ns	−0.658**	−0.742**	ns	ns	ns	ns

*P < 0.05; **P < 0.01. CI, channel index; EI, enrichment index; MI, maturity index; PPI, plant-parasitic index; ns, not significant; SI, structure index.

In our study, the Ba_1,2_ genera *Rhabditis*, *Mesorhabditis, Cephalobus*, and *Plectus*; the Fu_2,4_ genera *Aphelenchoides* and *Dorylaimoides*; the Om_4,5_ genera *Eudorylaimus*, *Mesodorylaimus*, and *Aporcelaimellus*; and the P_4,5_ genera *Mylonchulus*, *Prionchulus*, and *Oxydirus* were the most sensitive to higher soil contents of Pb, Zn, and As. In contrast, Pp_3_ obligate plant parasitic nematodes did not appear to suffer from the effects of contamination by Pb, As, and Zn in the highly polluted meadows.

Our findings partially agree with those of previous investigations of the impacts of heavy metals on particular nematode taxa but contradict with some data. For example, Korthals *et al*. (2000) reported high sensitivities of *Alaimus* (Ba_4_), *Thonus* (Om_4_), and *Aporcelaimellus* (Om_5_) and the tolerance of *Aphelenchoides* (Fu_2_), *Tylenchorhynchus*, *Rotylenchus*, and *Pratylenchus* (Pp_3_) to experimental additions of Cu and Zn to the soil. Smit *et al*. (2002) observed the dominance of *Aphelenchoides* and *Mesodorylaimus* (Om_4_) in soils that received the highest amounts of Zn in comparison to lower amounts. Sánchez-Moreno *et al*. (2006) found that *Alaimus*, *Boleodorus*, and *Tetylenchus* (Pp_2_); *Criconemoides*, *Hemicycliophora*, *Heterodera*, and *Hoplolaimus* (Pp_3_); and *Monhystera* (Ba_2_), *Panagrolaimus* (Ba_1_), and *Dorylaimus* (Om_4_) were the most sensitive to contamination with Pb, Cu, Zn, and Ni in an affected riparian zone 2 yr after a large mine spillage. Similarly, Pen-Mouratov *et al*. (2008) reported that the abundances of *Chiloplacus*, *Plectus*, *Dorylaimus*, *Eudorylaimus*, and *Nygolaimus* (P_5_) and *Aphelenchoides*, *Tylenchorhynchus*, *Pratylenchus*, and *Tylenchus* (Pp_2_) were negatively correlated with As, Cu, Zn, and Pb pollution. Rodríguez-Martín *et al*. (2014), however, reported that *Aphelenchoides* and *Aphelenchus* (Fu_2_); *Eudorylaimus* and *Ecumenicus* (Om_4_); *Nygolaimus*, *Discolaimus*, and *Discolaimoides* (P_4_); and *Helicotylenchus* were tolerant to high Pb contents. These contradictory findings for the sensitivity *vs*. tolerance of particular nematode taxa to heavy-metal loads among previous studies and our findings were probably due to differences in the heavy metals and their combinations in the soils. The total contents of the elements measured in these studies were also highly variable, which may also have been an important factor contributing to the contradictory results. For example, iron (Fe) in a study by Martinez *et al*. (2018) was the main soil pollutant in a mining area in the southern Philippines (2,300–2,600 mg/ kg), where Pb and Zn contents were very low (20– 60 mg/kg). This study found that some genera of predacious and omnivorous nematodes were most abundant at sites with elevated heavy-metal contents, especially Fe, even though these nematodes were generally expected to be sensitive to both chemical pollution and physical disturbance (e.g., *Ironus* and *Eudorylaimus*). In contrast, the Pb and Zn contents in our study reached maxima of 4,030 mg/kg and 3,867 mg/kg, respectively, at the heavily polluted sites. The genera *Eudorylaimus*, *Mesodorylaimus*, and *Aporcelaimellus* (Om_4-5_) were only sporadically present at the sites with lower amounts of Pb and Zn, suggesting that the Pb and Zn contents were negatively correlated with the abundances of these Om nematodes.

The lower abundances of the majority of the taxa, and the absence of several taxa, at the polluted sites than the control site indicated changes in the community structures and led to changes in the community indices. The abundances of these taxa were significantly lower at the most heavily polluted sites, and reductions of these indices were less pronounced with increasing distance from the sources of pollution. Our results are in accordance with the findings by Sánchez-Moreno and Navas (2007), Shao *et al*. (2008), Park *et al*. (2011), and Šalamún *et al*. (2014). These studies also found that heavy-metal pollution strongly affected the structures of the soil nematode communities, indicated by lower values of the maturity indices (MI and MI2–5) or SI. In contrast, EI in two of these studies (Park *et al*., 2011; Šalamún *et al*., 2014) did not differ significantly between polluted and unpolluted sites, suggesting that heavy-metal contamination may have disturbed the soil ecosystem by suppressing biological activity.

Our findings for obligate plant parasitic nematode taxa were consistent with the results reported by Shao *et al*. (2008) from mine tailings 12 yr after dwindling yields caused the cessation of Pb and Zn mining in 1996. This study also found that plant-feeding nematodes were not negatively affected by heavy-metal pollution and that their abundance increased with the recovery of vegetation at disturbed sites, consistent with the findings by Háněl (2004) from waste dumps of industrial pyrite and calcium. The abundance of plant-feeding nematodes is mostly determined by community structure, biomass, and the vigor of plants (Bongers and Ferris, 1999). The sites investigated in our study were typical alluvial meadows with no physical injuries and dominated by various species of grasses and herbs, suggesting that the effects of heavy metals on plant-feeding nematodes can be mitigated by the presence of plants with rich root systems that serve as sources of food. In contrast, Park *et al*. (2011) reported that the abundance of plant-feeding nematodes was considerably lower at sites contaminated with heavy metals than at uncontaminated sites; the species of plants at the study sites differed little, but their growth was poorer at the contaminated than the uncontaminated sites.

## Conclusion

The relatively large number of nematode taxa recorded in the alluvial meadows of the Litavka River demonstrated the capacity of local soils to harbor diverse nematode fauna in this area. Some of these soils, however, have been affected by heavy metals from ponds that sediment mine waste and from waste heaps, spread repeatedly by contaminated flood waters, and our analyses indicated that these pollutants have adversely affected the community of soil nematodes at the target sites. Pb, Zn, As and, to a lesser extent, Cu were the main pollutants responsible for the observed impact. Nematode abundance, number of taxa, and consequently nematode biomass, as well as the trophic complexity of the nematode communities, were significantly reduced at the heavily polluted sites. Interestingly, the abundances of all functional guilds among the bacterivores (Ba_1,2,3,4_), predators (P_3,5_), omnivores (Om_4_,_5_), plant parasites (Pp_2_), and fungivores (Fu_2,4_) were sensitive to heavy metals, and the abundance of Pp_3_ nematodes did not indicate heavy-metal contamination. H´gen, MI, MI2–5, EI, SI, and BI were the most valuable community indices for assessing the impact of heavy metals. Our results thus support the general view that communities of soil nematodes provide highly responsive metrics and sensitivities as bioindicators of heavy metal pollution in the environment.
